# Integrated multi-omics to elucidate the interplay between rumen microorganisms and host metabolism in Hu sheep supplemented with herbal preparations

**DOI:** 10.1128/msphere.00024-25

**Published:** 2025-03-13

**Authors:** Chunhui Wang, Qiao Li, Xingcai Qi, Huihui Wang, Yi Wu, Keyan Ma, Juanjuan Song, Zilong Liu, Youji Ma

**Affiliations:** 1 College of Animal Science and Technology, Gansu Agricultural University, Lanzhou, China; 2 Gansu Key Laboratory of Animal Generational Physiology and Reproductive Regulation 74661 , Lanzhou, China; University of Wisconsin-Madison, Madison, Wisconsin, USA

**Keywords:** rumen microorganisms, rumen metabolome, serum metabolome

## Abstract

The aim of this experiment was to study the effects of herbal preparations on serum metabolites, rumen microorganisms, and their metabolites, and the relationship between them. Hu sheep ram lambs with an average initial weight of (19.57 ± 1.56 kg) at 3 months of age were selected and randomly divided into three groups of six lambs each. The groups were as follows: the control group (Con), which was fed the basic diet; Test I, which was fed a diet with 0.5% herbal preparations added to the concentrate; and Test II, which was fed a diet with 1% herbal preparations added to the concentrate. Also, the main component of herbal medicine is polysaccharide. The pre-experimental period was 10 days and the experimental period was 90 days. The results of the study showed that the addition of herbal preparations resulted in differences in species, abundance, and metabolic functions of rumen microorganisms. The abundance of rumen-dominant bacteria, such as Firmicutes and Proteobacteria, increased after the addition of herbal preparation, which was more conducive to rumen development. In addition, after the addition of 0.5% herbal preparation, there was an increase in the abundance of fermenting carbohydrate (CHO) and fiber-degrading bacteria (e.g., *Ruminococcus* and *Prevotella*). Herbal preparations significantly altered rumen microorganisms and serum metabolite compositions. Metabolites such as bile acids, L-glutamine, cytosine, and choline, which contribute to the antiviral and anti-inflammatory effects, nutrient metabolism, and immune responses, and increased rumen microbial activity, were increased in the rumen of the experimental group with the addition of the herbal preparations. The increase in serum metabolites, such as L-tryptophan, and the pathways of tryptophan metabolism and glutathione metabolism in animals were also significantly higher than those in Con. *Prevotella* and *Ruminococcus* were significantly positively correlated with histamine and L-arginine. The *uncultured_rumen_bacterium* was significantly negatively correlated with serum metabolites testosterone and guanine, but *Prevotella* and *Ruminococcus* were significantly positively correlated with both metabolite testosterone and guanine.

**IMPORTANCE:**

In this study, we investigated the changes in rumen microbes after supplementation with herbal preparations. We used 16S sequencing and metabolomics approaches to explore changes in rumen contents and serum metabolites and their interrelationships. Our findings revealed marked changes in rumen microbial profiles, including changes in species composition, abundance levels, and metabolic activities induced by herbal supplementation. The increased abundance of beneficial bacteria (e.g., fixative and proteobacteria) in the rumen was more favorable for their survival and colonization of the rumen. In addition, a surge in the abundance of fermenting carbohydrate and fiber-degrading bacteria was observed. It was also found that the addition of herbal preparations enhanced antiviral and anti-inflammatory responses, nutrient metabolism, immune function, and stimulation of rumen microbial activity as well as facilitated the acceleration of body metabolism in Hu sheep.

## INTRODUCTION

Ruminants primarily rely on the rumen for food digestion, and the rumen microbial community plays a crucial role in nutrient metabolism, absorption, and immune functions related to the intestines (1). Changes in dietary composition and nutritional factors can regulate the composition and abundance of rumen microorganisms. Studies have shown that rumen microorganisms ferment carbohydrates, proteins, and fats in feed into forms of energy that animals can utilize (2). Rumen microorganisms are essential for ruminant digestion, as they ferment fibers and other organic compounds that are indigestible by mammals, synthesizing non-protein nitrogen. In addition, rumen microorganisms can break down dietary fiber substances that ruminants cannot utilize into volatile fatty acids, which serve as energy sources. These microbes can provide 70% of the daily energy needs of ruminants (3). Therefore, understanding the composition and function of the rumen microorganisms community is crucial for improving the digestive efficiency and productivity of ruminants.

The composition of rumen microorganisms affects the digestion and metabolism of ruminants, as well as changes in the content of metabolites. Metabolites present in rumen fluid play a critical role in biological metabolism, growth, nutrient utilization by the host, and overall health status. These metabolites significantly influence the growth of ruminants (4). Blood serves as an intermediary biological fluid, containing metabolites excreted from the animal body and facilitating communication between various organs and tissues (5). Therefore, the metabolism of compounds in the blood plays a crucial role in the growth and development of animals. Research indicates that the impact of rumen microbial metabolites and serum metabolites on milk production yield (MPY) surpasses that of rumen microbial composition and function (6). Enhancing the microbiome and host dependency of MPY involves optimizing milk quality and quantity through alterations in rumen microflora via dietary management or genetic selection strategies (7). The metabolic products of tryptophan can improve the function of the rumen, increasing the efficiency of ruminal fermentation and the microbial composition (8). Furthermore, recent studies have indicated that the rumen microbiota influences methane emissions and feed efficiency in dairy cows in conjunction with the host metabolome (9, 10). Clinical studies have also shown that a large amount of D-glucose is directly transported from the rumen contents to the blood (11). Therefore, a correlation exists between rumen contents metabolism and serum metabolism.

Herbal preparations as feed additives have been widely used in animal husbandry, particularly showing unique advantages in improving animal health, enhancing immunity, improving digestion efficiency, and promoting growth (12). Chinese herbal medicine (Sauce llorón extract) shows promise as an antibiotic alternative for enhancing animal productivity and rumen function (13). The supplementation of Chinese herbal medicine in animal diets has been shown to enhance animal growth, boost immunity, and exhibit minimal toxic or side effects, making it a preferred choice (14). Research has shown that feeding herbal preparations to beef cattle at a dosage of 20 g/kg produced significant results, not only in terms of average daily gain, but also in terms of digestive enzyme activity and overall digestibility (15). Wang et al. (16) found that supplementing astragalus root during feeding can increase the average daily gain of Tibetan sheep. At the same time, studies have found that astragalus polysaccharides can improve the growth performance and intestinal development of livestock and poultry (17).

Nevertheless, limited research has been conducted on the distinct metabolites and associated metabolic pathways in rumen microorganisms, rumen contents, and serum following the supplementation of Chinese herbal medicine preparations in animal diets. While numerous studies have explored the rumen microbial community and metabolism, there remains a scarcity of data regarding microbial functional groups and metabolites. Furthermore, the correlation between rumen microbiota, rumen metabolome, and host serum metabolome remains predominantly unexplored. Consequently, the aim of this study was to investigate the interaction between rumen microorganisms and rumen as well as serum metabolism in Hu sheep after the addition of herbal preparations.

## MATERIALS AND METHODS

### Experimental animals and materials

The feeding trial was conducted at Longqin Modern Agriculture and Animal Husbandry Industry Co., Ltd. in Gulang County, Wuwei City, Gansu Province, spanning from March to June 2023. The Chinese herbal medicine formulations consist primarily of 11 traditional Chinese medicinal ingredients, including *Codonopsis pilosula* (26%), medicated leaven (20%), malt (13%), stir-fried hawthorn (13%), *Astragalus membranaceus* (4%), *Poria cocos* (4%), *Atractylodes macrocephala* (4%), cardamom (4%), *Citrus reticulata* Blanco (4%), hyacinth bean (4%), and *Schisandra* (4%). The nutrient contents of the Chinese herbal preparations were as follows: crude protein (CP) 11.84%, ash 11.17%, neutral detergent fiber (NDF) 23.69%, acid detergent fiber (ADF) 11.26%, Ca 0.97%, and P 1.94%. The effective active components in the herbal preparations include water extracts crude extract at 33.86%, total polysaccharides at 27.21%, and total flavones at 0.36%. The diet and experimental animals were supplied by Longqin Modern Agriculture and Animal Husbandry Industry Co., Ltd. in Gansu Province. Detailed information regarding the composition and nutritional content can be found in the accompanying Table 1.

**TABLE 1:** Base diet composition and nutritional level (feeding basis)

Ingredients	Content (%)
Corn	40
Cottonseed meal	5.5
Soybean meal	12.5
Peanut seedling	29
Wheat bran	9
CaHPO_4_	0.5
NaCl	1
Limestone	1.5
Premix^*a*^	1
Total	100
Nutrient levels^*b*^	
ME	8.57
CP	14.51
EE	2.21
NDF	30.11
ADF	19.58
Ca	1.01
P	0.47

^*a*^ The premix provided the fllowing per kg of diets: Fe, 75 mg; Cu, 14 mg; Zn, 80 mg; Se, 0.25 mg; I, 0.25 mg; Co, 0.3 mg; Mn, 400 mg; VA, 3,000 IU; VD, 500 IU; VE 200 IU.

^*b*^ Measured values; ME, metabolizable energy; EE, ether extract.

### Experimental design and feeding management

In this experiment, a single-factor randomized design was employed to select male Hu sheep lambs with an average initial weight of 19.57 ± 1.56 kg at 3 months of age. The animals were randomly assigned to three groups, each consisting of six individuals. The groups were as follows: the control group (Con), which was fed the basic diet; Test I (T1), which was fed a diet with 0.5% herbal preparations added to the concentrate; and Test II (T2), which was fed a diet with 1% herbal preparations added to the concentrate. The experiment lasted a total of 100 days, which included a pre-experimental phase of 10 days and a formal testing period of 90 days. Prior to the commencement of the trial, the Hu sheep were immunized according to the prescribed protocol, and the housing facility was thoroughly cleaned and disinfected. During the experiment, the sheep had free to access feed and water.

### Sample collection

On the final day of the feeding trial, blood samples were obtained in the early morning from the test sheep while they were in a fasting state. The blood was collected from the jugular vein of each sheep into a coagulation-promoting vessel using a vacuum blood collection tube. The serum samples were incubated at 4°C for 30 minutes, followed by centrifugation at 1,500 rpm for 10 minutes. The resulting serum was then carefully transferred into a cryovial and stored at −80°C to facilitate subsequent analysis of the serum metabolome.

At the conclusion of the feeding trial, all test lambs were euthanized following a 12 hour fasting period. Subsequently, 10 mL of rumen contents was extracted from each lamb, divided into three sterile freezing tubes, rapidly frozen using liquid nitrogen, and then stored at −80°C. These samples will be utilized for assessing microbial diversity and conducting metabolomic analysis.

### Indicator measurement methodology

#### Determination of microbial indicators

Using the TGuide S96 Magnetic Soil/Stool DNA Kit (Tengen Biochemical Technology [Beijing] Co., Ltd.), genomic DNA was extracted from rumen contents according to the manufacturer's instructions. The V1~V9 highly variable region of the 16S rRNA gene was amplified with primers (27F: AGRGTTTGATYNTGGCTCAG; 1492R: TASGGHTACCTTGTTASGACTT). After quantification of the amplicons, standardized equimolar concentrations of the amplicons were mixed and sequenced on the PacBio Sequel II platform (Beijing BioTech Co., Ltd., Beijing, China).

#### Metabolomics analysis

The serum and rumen content were thawed on ice and vortexed for 10 seconds. Subsequently, regarding serum samples, 50 µL of sample and 300 µL of extraction solution acetonitrile [CH_3_CN; ACN]:methanol = 1:4, vol/vol) containing the internal standard were combined in a 2 mL centrifuge tube. The mixture was vortexed for 3 minutes and then centrifuged at 12,000 rpm for 10 minutes at 4°C. After centrifugation, 200 µL of the supernatant was collected. For the rumen content, 20 mg was taken and 400 µL of internal standard solution (methanol:water = 7:3, vol/vol) was added. The sample was vortexed for 3 minutes, sonicated for 10 minutes, and then vortexed for 1 minute in an ice bath. Both samples were allowed to stand for 30 minutes at −20°C. The serum sample was further centrifuged at 12,000 rpm for 10 minutes at 4°C, but the rumen content samples were centrifuged at the same speed for 10 minutes at 4°C. Finally, 200 µL of the sample supernatant was analyzed using a liquid chromatograph mass spectrometer (LC-MS).

### Data statistics and analysis

Eligible sequences with a similarity threshold greater than 97% were assigned to an operational taxonomic unit (OTU) using USEARCH (version 10.0). OTUs/ASVs (amplicon sequence variants) were taxonomically annotated using the SILVA database (18) (release 138.1) based on the naïve Bayesian classifier in QIIME2 with a confidence threshold of 70% (18–20). Alpha analysis was performed using QIIME2 software to determine the diversity of species within the samples. The Chao1 and Ace indices are utilized to assess species richness, which refers to the total count of species. In contrast, the Shannon and Simpson indices are employed to quantify species diversity. Beta diversity was calculated by principal coordinate analysis (PCoA) to assess the diversity of species between the samples. Permutational multivariate analysis of variance (PERMANOVA) was employed to assess the significance of beta diversity differences among samples across various groups. In the OPLS-DA，the coefficient *R*^2^*X* quantifies the proportion of inter-group variation explained by the model, with higher values suggesting a greater explanatory power and consequently more pronounced group differences. A statistically significant result is inferred when the *P*-value <0.05, which denotes high reliability of the test outcome. Differential abundance taxa were assessed using linear discriminant analysis (LDA) combined with effect size values (LEfSe) (21). Analysis of ruminal microbial sequencing data (bacterial abundance and diversity were compared) were using the online platform BMKCloud (https://www.biocloud.net). Utilizing PICRUSt2, an evolutionary tree was constructed by aligning the signature sequences (16S rRNA) with the reference sequences in the Integrated Microbial Genomes database. This approach facilitated the identification of the closest related species to the signature sequences, enabling the prediction of gene information for unknown species based on the gene type and abundance data of known species. Furthermore, integration of the KEGG pathways with gene-specific KEGG pathway information allowed for the prediction of community-wide pathways (22). The functional abundance of functional stratum 2 in this sample was analyzed using the G-TEST in STAMP, which employs a two-by-two *t*-test to compare different groups with a significance threshold of 0.05 (*P* < 0.05 indicating significance).

Unsupervised principal component analysis (PCA) was performed by statistics function prcomp within R (https://www.r-project.org/). The data were unit variance scaled (is Z-score standardization) before unsupervised PCA. For two-group analysis, differential metabolites were determined by variable importance in projection (VIP) (VIP > 1) and *P*-value (*P*-value <0.05, Student’s *t*-test). The VIP values were derived from the OPLS-DA model (with biological replicates ≥ 3)，which also contains score plots and permutation plots, generated using R package MetaboAnalystR (23). The data were log transform (log_2_) and mean centering before OPLS-DA. In order to avoid overfitting, a permutation test (200 permutations) was performed. Identified metabolites were annotated using KEGG Compound database (http://www.kegg.jp/kegg/compound/), and annotated metabolites were then mapped to KEGG pathway database (http://www.kegg.jp/kegg/pathway.html). The pathways mapped by significantly regulated metabolites were then fed into metabolite sets enrichment analysis (MSEA). The significance of the enrichment was determined by the *P*-values from the hypergeometric test. The closer the *P*-value is to 0, the more significant the enrichment is.

## RESULTS

### Rumen microorganisms

A total of 235,940 CCS (circular consensus sequencing) sequences were obtained from 18 samples of rumen contents that were sequenced and identified using barcode methods. Each sample produced a minimum of 12,003 CCS sequences, with an average yield of 13,108 CCS sequences per sample. The effective tags of all the samples were clustered with 97% concordance, and a total of 1,710 OTUs were obtained (Table S1). Representative sequences of OTUs were selected for species annotation, and a total of 22 phyla, 33 orders, 64 orders, 125 families, 248 genera, and 389 species were annotated in all the rumen content (Table S2).

**Fig 1:**
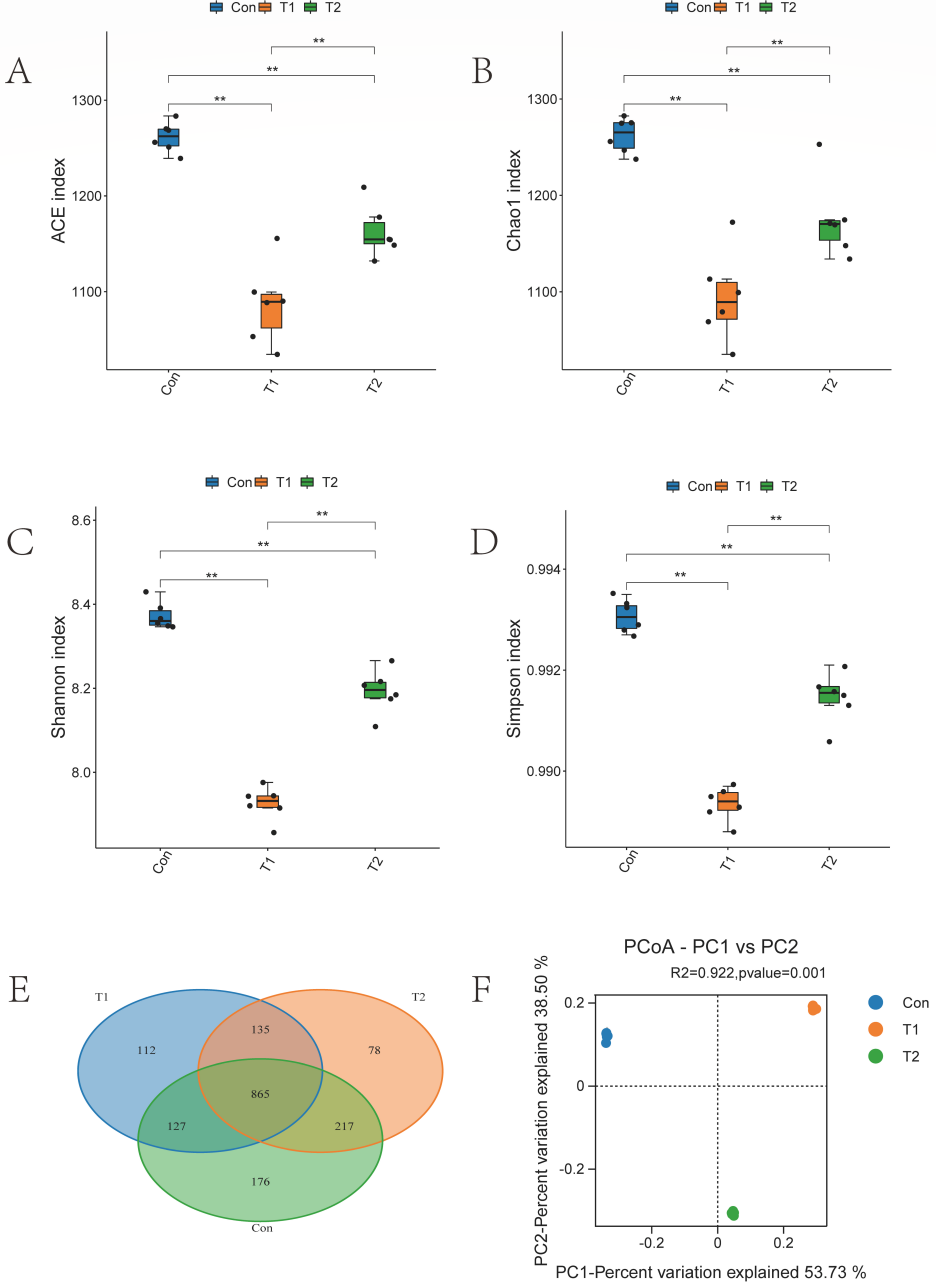
Sequencing results and statistical analysis of diversity. (A–D) The Simpson, Shannon, Chao1, and ACE indices for the three test groups. Adjusted *P < 0.05 and **P < 0.01 by Student’s *t*-test. Rumen microorganisms (E) Venn and rumen microorganisms (F) PCoA based on all samples. Different colors of Venn and PCoA represent different sample groups.

Alpha diversity analysis based on OTUs from rumen microbial 16S rRNA gene sequencing results revealed that ACE, Chao1, Shannon, and Simpson indices were significantly lower (*P* < 0.05) in both T1 and T2 groups compared to the Con (Fig. 1A through D). This suggests that the inclusion of herbal preparations led to a decrease in the abundance and diversity of rumen microbiota. The Venn diagram illustrating the OTUs across the three experimental groups is presented in Fig. 1E, indicating a total of 1,710 OTUs, with 865 OTUs shared among all groups, 176 unique OTUs in the Con, 112 unique OTUs in the T1 group, and 78 unique OTUs in the T2 group. PCoA based on Bray-Curtis dissimilarity at the OTU level was conducted for the three experimental groups, and PERMANOVA was employed to assess the overall significance among the groups. The results were 53.73% and 38.50% for principal component (PC1) and PC2, respectively, as shown in Fig. 1F. Significant differentiation was observed between the Control, T1, and T2 groups (*R*^2^ = 0.992, *P* < 0.05), indicating a substantial impact of herbal preparations on the rumen microbial composition in sheep.

**Fig 2:**
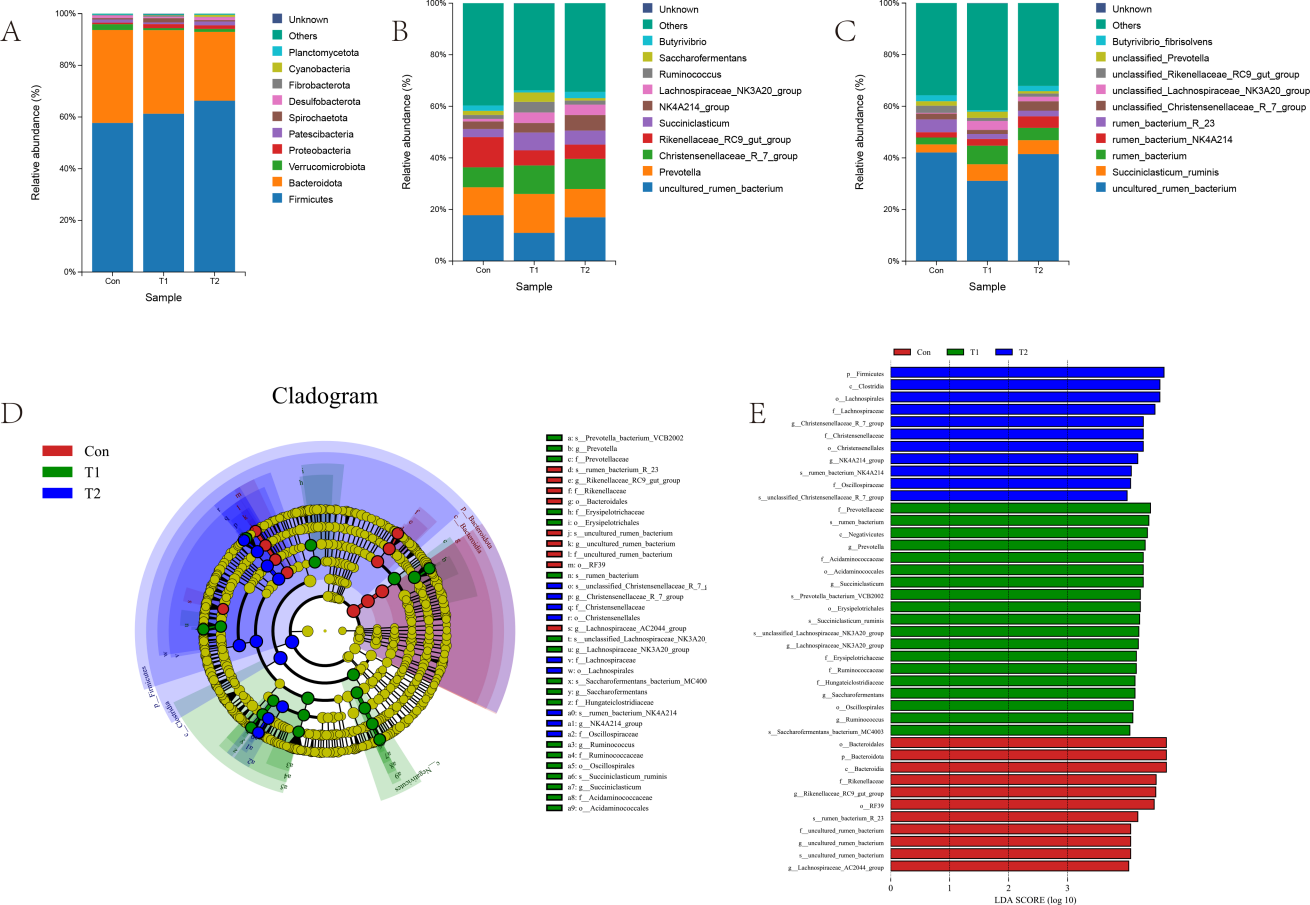
(**A**) The phylum-level microbial composition of the rumen of the three experimental groups. (**B**) The genus-level microbial composition of the rumen of the three experimental groups. (**C**) The species-level microbial composition of the rumen of the three experimental groups. (**D**) The LEfSe analysis cladogram of rumen microorganisms. (**E**) LDA was used to estimate the effect of each component (species) abundance on the difference. LDA > 4, *P* < 0.05.

The distribution of rumen microbiota at the phylum, genus, and species levels is illustrated in the Fig. 2. At the phylum level, Firmicutes and Bacteroidota were identified as the predominant phylum in the rumen of Hu sheep, with their relative abundance showing variations following the introduction of herbal preparations. Specifically, the relative abundances of Firmicutes, Proteobacteria, and Cyanobacteria were higher in the T2 and T1 groups compared to the Con post-herbal supplementation. Conversely, the relative abundances of Bacteroidota and Verrucomicrobiota were higher in the Con than in the other experimental groups, while Spirochaetota exhibited the highest relative abundance in the T1 (Fig. 2A). At the genus level, the relative abundances of *Lachnospiraceae_NK3A20_group*, *NK4A214_group*, *Succiniclasticum,* and *Christensenellaceae_R_7_group* surpassed those of the Con in both T1 and T2 groups. In contrast, the relative abundance of *Rikenellaceae_RC9_gut_group* decreased following herbal supplementation, and the relative abundances of *Prevotella*, *Saccharofermentans*, and *Ruminococcus* spp. were higher in T1 compared to the other experimental groups (Fig. 2B). At the species level, the relative abundance of *Succiniclasticum_ruminis*, *unclassified Lachnospiraceae_NK3A20_group*, *rumen_bacterium_NK4A214*, and *rumen_bacterium* exhibited higher levels in the T1 and T2 groups compared to Con following the administration of herbal supplements. Their relative abundance surpassed that of the Con. Conversely, the relative abundance of *rumen_bacterium_R_23* and *unclassified_Prevotella* decreased in both T1 and T2 groups. These findings indicate significant alterations in rumen microbial composition subsequent to the inclusion of herbal supplements in the diet, as depicted in Fig. 2C. Based on the results of the cluster analysis, we performed LEfSe analyses on the Con, T1, and T2 groups. The Con group Bacteroidales, Bacteroidota, *Rikenellaceae_RC9_gut_group,* RF39, *rumen_bacterium_R_23*, *Lachnospiraceae_AC2044_group,* and *uncultured_rumen_bacterium* were significantly higher in relative abundance than the other two groups. The T1 group *Prevotella*, *rumen_bacterium*, *Succiniclasticum_ ruminis*, *Succiniclasticum,* and 19 other bacterial species were significantly more abundant than the other two groups. The T2 group Firmicutes, Christensenellaceae, *rumen_bacterium_NK4A214,* and 11 other bacterial species were significantly more abundant than the other two groups (LDA > 4, *P* < 0.05; Fig. 2D and E).

**Fig 3:**
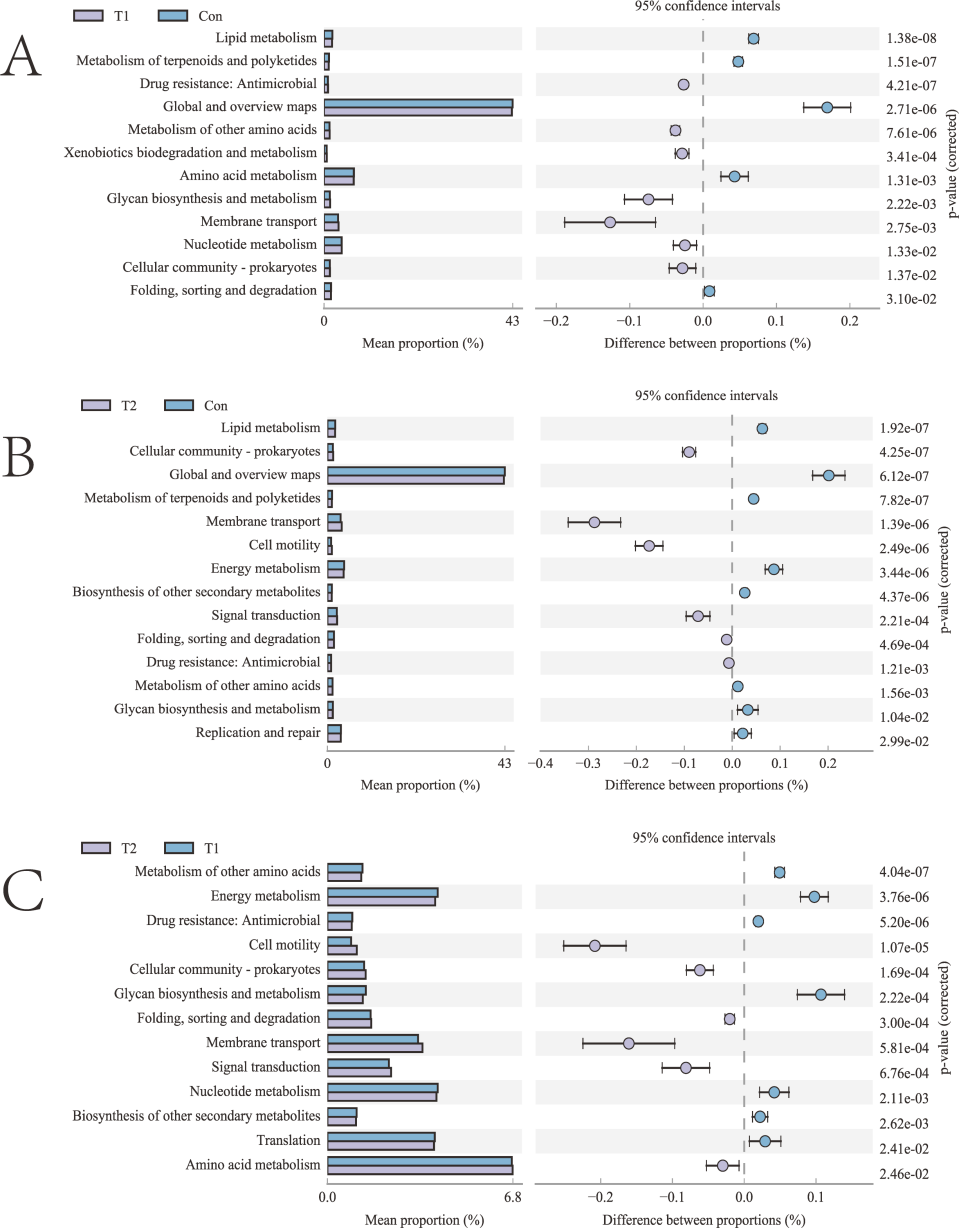
Differential analysis of KEGG metabolic pathways graph. (**A**) T1 vs Con, (**B**) T2 vs Con, (**C**) T2 vs T1.

The PICRUSt2 results indicate that functions related to lipid metabolism, metabolism of terpenoids and polyketides, as well as global and overview maps were significantly more abundant in the Con compared to the T1 and T2 groups (*P* < 0.05). Moreover, functions associated with metabolism of other amino acids and glycan biosynthesis and metabolism were notably higher in the T1 compared to the Con and T2 groups (*P* < 0.05). On the other hand, cell motility and signal transduction functions exhibited significantly higher levels in the T2 than in the T1 and Con (*P* < 0.05). Conversely, energy metabolism and biosynthesis of other secondary metabolites were significantly lower in the T2 than in the other two groups (*P* < 0.05). Regarding drug resistance, functions related to antimicrobial and membrane transport were significantly lower in the Con compared to the other experimental groups. Specifically, for drug resistance: for antimicrobial, the T1 group was significantly higher than the T2 group, while for membrane transport, the T2 group was significantly higher than the T1 group (*P* < 0.05, Fig. 3A through C).

### Metabolomes of rumen contents

Samples of rumen contents from different experimental groups showed a significant separation between rumen contents. PC1 and PC2 explained 48.13% and 22.94% of the variance, respectively. This indicates substantial variability in rumen metabolites across the experimental groups, while showing less difference among replicates within each group (Fig. S1A).

In this model, *R*^2^*X* and *R*^2^*Y* represent the explanatory power of the X and Y matrices, respectively, by the constructed model, while *Q*^2^ signifies the predictive capability of the model. Various experimental groups including T1 vs Con, T2 vs Con, and T2 vs T1 in Hu sheep were analyzed using the OPLS-DA technique. The results demonstrated robust group clustering and significant inter-group distinctions (Fig. S1B through C). As depicted in Fig. S1E through G, the *R*^2^*Y* values for T1 vs Con, T2 vs Con, and T2 vs T1 were 1, 1, and 1, respectively, with corresponding *Q*^2^ values of 0.987, 0.995, and 0.992. These findings indicate a well-fitted and predictive OPLS-DA model suitable for subsequent data analysis. Differential metabolite groups were further identified based on OPLS-DA criteria, including fold change (FC) ≥ 2 or FC ≤ 0.5, *P* < 0.05, and VIP > 1 . The outcomes of the screening were visually represented using volcano plots and k-means plots.

**Fig 4:**
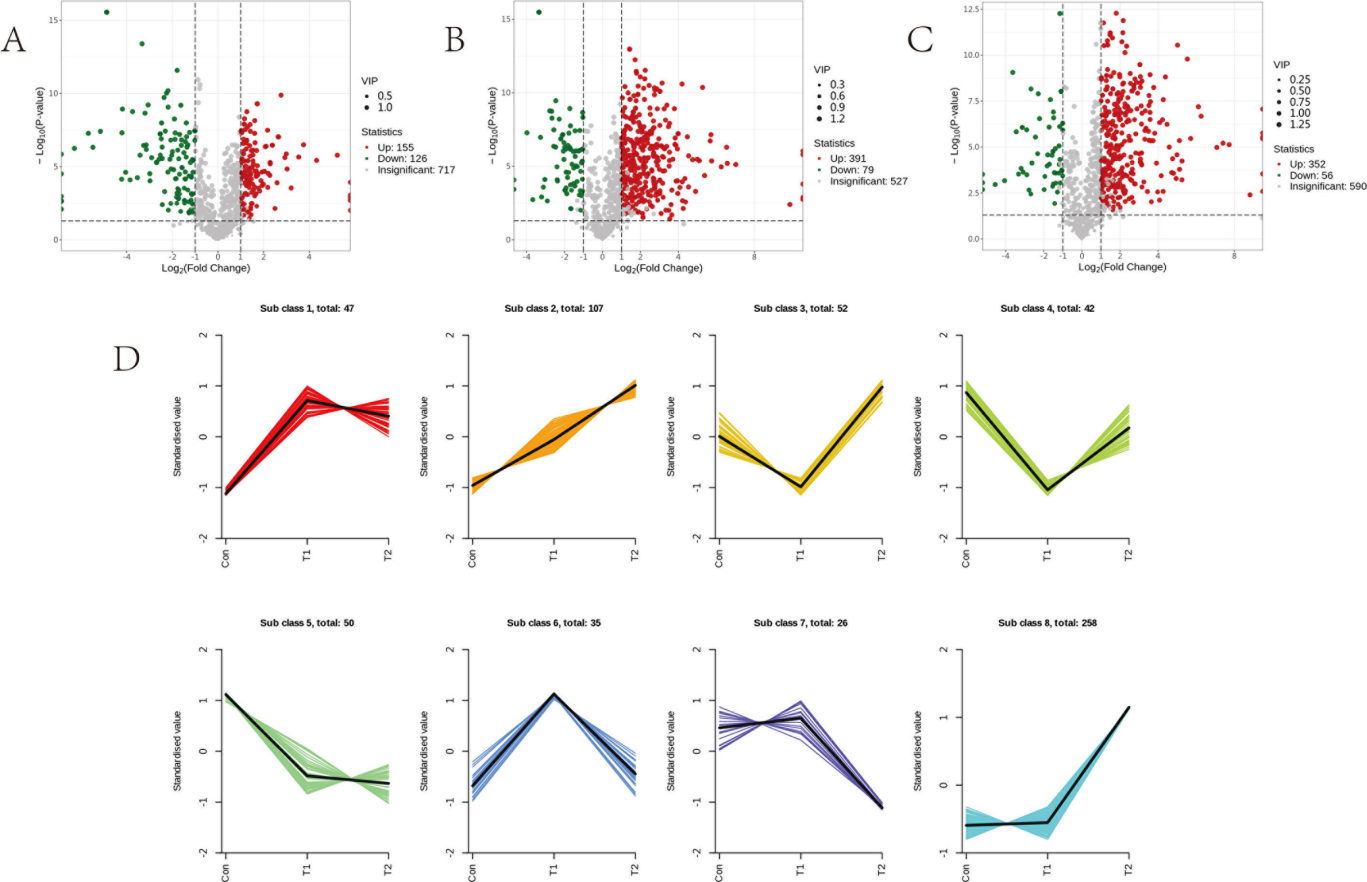
Differentially accumulated metabolites among different experimental groups. (**A**) Volcano plots of differential metabolites between T1 vs Con, (**B**) volcano plots of differential metabolites between T2 vs Con, and (**C**) volcano plots of differential metabolites between T2 vs T1. (**D**) Metabolite variation tendencies among the eight cluster profiles.

In order to investigate the variation of metabolites in the rumen of sheep among different experimental groups, we analyzed the differential metabolites accumulated between T1 vs Con, T2 vs Con, and T2 vs T1. Our findings revealed a total of 281 differential metabolites (155 upregulated and 126 downregulated) were identified in T1 vs Con, 470 differential metabolites (391 upregulated and 79 downregulated) were identified in T2 vs Con, and a total of 408 differential metabolites (352 upregulated and 56 downregulated) were identified in T2 vs T1 (Fig. 4A through C). The six profiles showed clear clustering of metabolite changes among different groups, and the trends are shown in Figure. (Fig. 4D).

**Fig 5:**
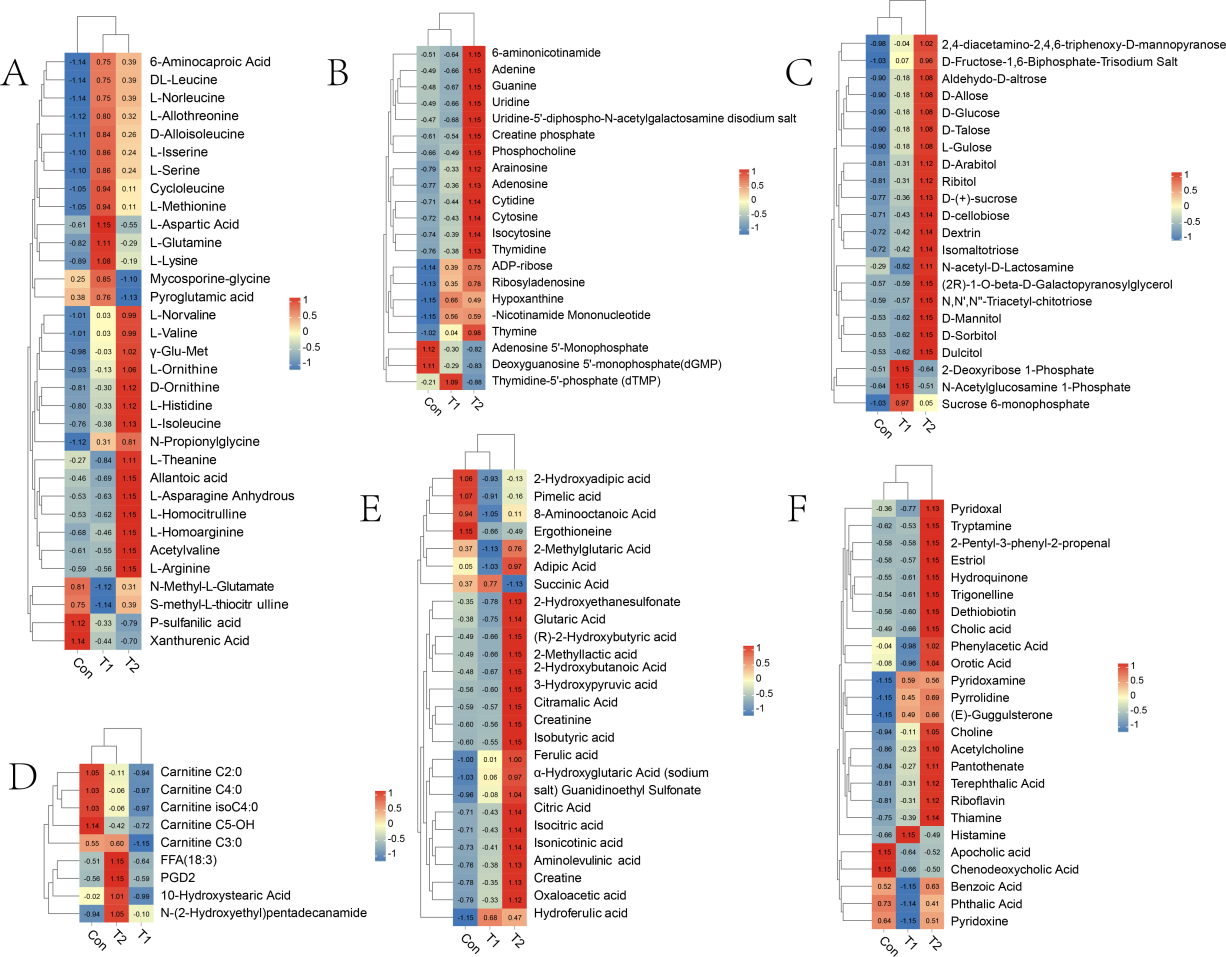
Distributions of accumulation profiles of differential metabolites detected during three experimental groups. (**A**) Amino acids and their derivatives; (**B**) nucleotide and its metabolites; (**C**) carbohydrates and its metabolites; (**D**) fatty acyl compounds (FA); (**E**) organic acid and its derivatives; (**F**) other metabolites.

The differential metabolites screened between the different groups were mainly categorized as amino acids and their metabolites, nucleotides and their metabolites, organic acids and their derivatives, carbohydrates and their metabolites, fatty acyls, coenzymes, and vitamins. To delve deeper into the variation of differential metabolites between groups in the rumen, heat map analysis of differential metabolites was performed. The results showed that nucleotides and their metabolites, carbohydrates and their metabolites, organic acids and their derivatives, bile acids, heterocyclic compounds, coenzymes, and vitamins in T1 and T2 groups showed an upward trend compared with Con (Fig. 5B, C, E, and F); amino acids and their derivatives and fatty acyl groups play different roles in the rumen due to different metabolites, so the significance of various metabolites in different groups is different. Specifically, after adding herbal preparations, the content of amino acids and their derivatives increased compared with Con (Fig. 5A). However, it should be noted that there was a general trend of increase in the content of fatty acyl upon the addition of 0.5% herbal preparation (Fig. 5D).

**Fig 6:**
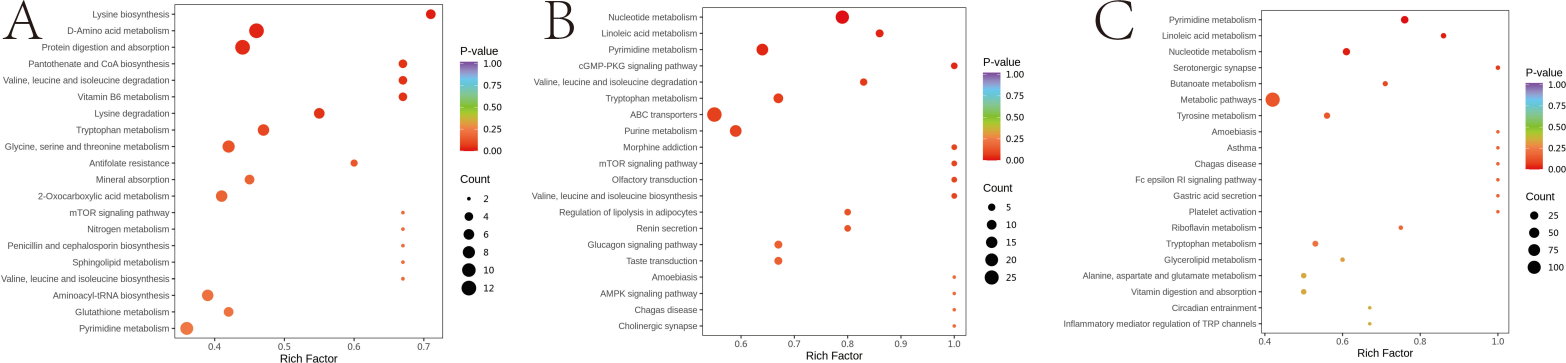
KEGG pathway enrichment analysis of rumen differentially expressed metabolites. The point color represents the *P*-value, and the size of the dots represents the number of differential metabolites. (**A**) T1 vs Con; (**B**) T2 vs Con; and (**C**) T2 vs T1.

To gain insights into the impact of different ratios of herbal preparations on the rumen metabolism in sheep, we conducted an enrichment analysis of differential metabolites. The results are shown in the Fig. 6, which illustrates that the differential metabolites identified between the two groups of T1 vs Con were mainly enriched in several pathways relevant to the animal. The top 10 of these enriched pathways were lysine biosynthesis, D-amino acid metabolism, protein digestion and absorption, pantothenate and coenzyme A (CoA) biosynthesis, valine, leucine and isoleucine degradation, Vitamin B6 metabolism, lysine degradation, tryptophan metabolism, glycine, serine, and threonine metabolism, and antifolate resistance (Fig. 6A). The top 10 metabolic pathways with differential metabolite enrichment between the T2 vs Con groups were nucleotide metabolism, linoleic acid metabolism, pyrimidine metabolism, cyclic guanosine monophosphate-protein kinase G (cGMP-PKG) signaling pathway, valine, leucine and isoleucine degradation, tryptophan metabolism, ABC transporters, purine metabolism, morphine addiction, and mTOR signaling pathway (Fig. 6B). Lastly, the top 10 metabolic pathways with differential metabolite enrichment between the two T2 vs T1 groups were pyrimidine metabolism, linoleic acid metabolism, nucleotide metabolism, serotonergic synapse, butanoate metabolism, metabolic pathways, tyrosine metabolism, amoebiasis, asthma, and Chagas disease (Fig. 6C).

### Serum metabolomics

The Fig. S2A illustrates obvious separation among serum samples from various experimental groups. PC1 and PC2 explained 27.89% and 15.49% of the variance, respectively. This indicates substantial variability in serum metabolites across the experimental groups, while showing less difference among replicates within each group.

The OPLS-DA technique was used to analyze the different experimental groups of Hu sheep serum T1 vs Con, T2 vs Con, and T2 vs T1. The results demonstrated robust group clustering and significant inter-group distinctions (Fig. S2B through D). The R^2^Y values for T1 vs Con, T2 vs Con, and T2 vs T1 were 0.999, 0.999, and 1, respectively, with corresponding Q^2^ values of 0.968, 0.968, and 0.928 (Fig. S2E through G). These findings indicate a well-fitted and predictive OPLS-DA model suitable for subsequent data analysis. Differential metabolite groups were further identified based on OPLS-DA criteria, including *P* < 0.05, and VIP > 1. The outcomes of the screening were visually represented using volcano plots and k-means plots, providing a clear and intuitive representation of the metabolic differences among the experimental groups

**Fig 7:**
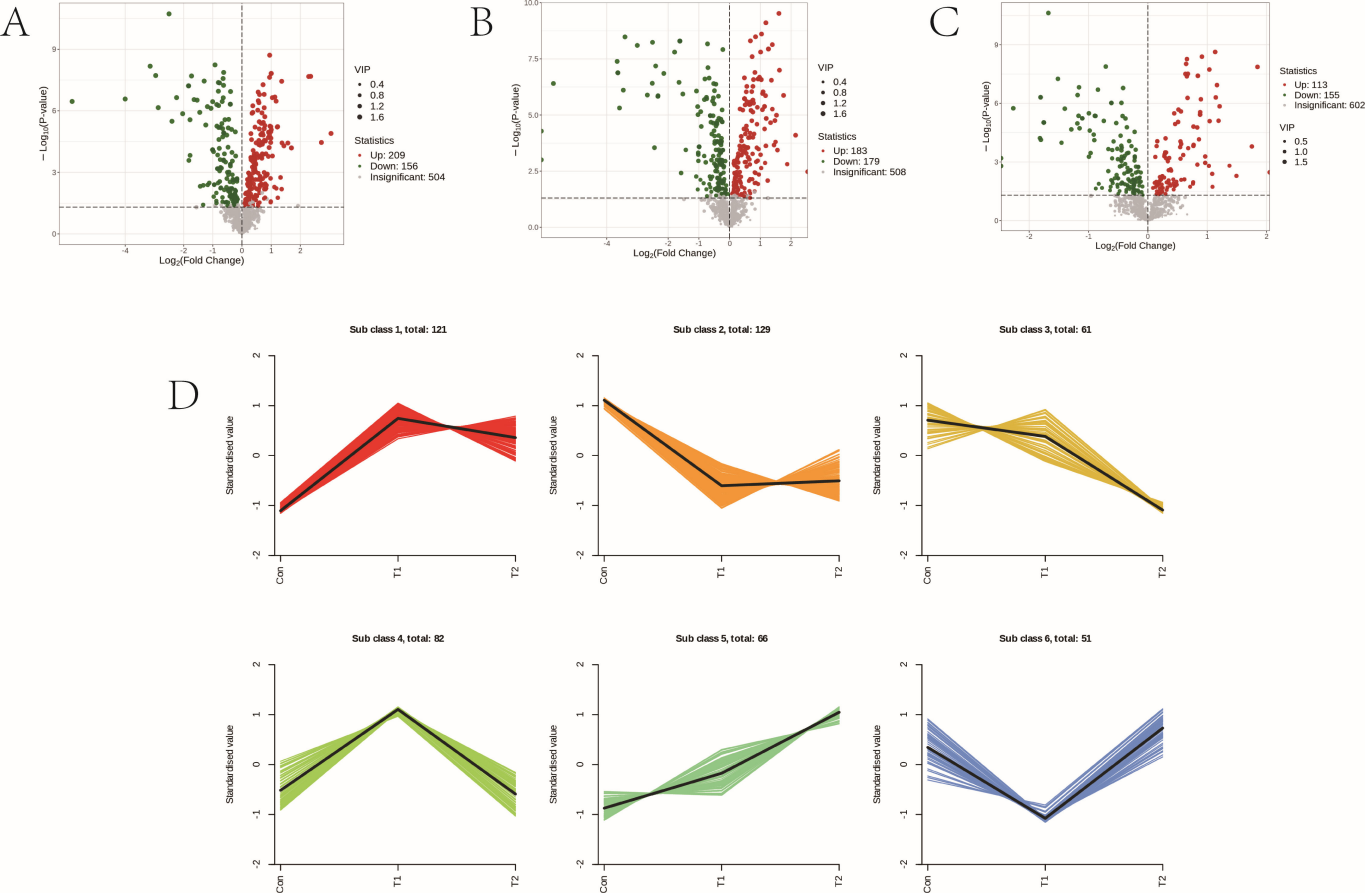
Differentially accumulated metabolites among different experimental group. (**A**) Volcano plots of differential metabolites among T1 vs Con, (**B**) volcano plots of differential metabolites among T2 vs Con, and (**C**) volcano plots of differential metabolites among T2 vs T1. (**D**) Metabolite variation tendencies among the six cluster profiles.

To investigate the changes in serum metabolites between the different experimental groups, we analyzed the differential metabolites accumulated between T1 vs Con, T2 vs Con, and T2 vs T1. Our analysis identified a total of 365 differential metabolites were identified in T1 vs Con, of which 209 were upregulated and 156 were downregulated, 362 differential metabolites were identified in T2 vs Con, of which 183 were upregulated and 179 were downregulated, and 268 differential metabolites were identified in T2 vs. T1, of which 113 were upregulated and 155 were downregulated (Fig. 7A through C). These six profiles showed a distinct clustering of metabolite changes between groups, and the trends are shown in Fig. 7D.

**Fig 8:**
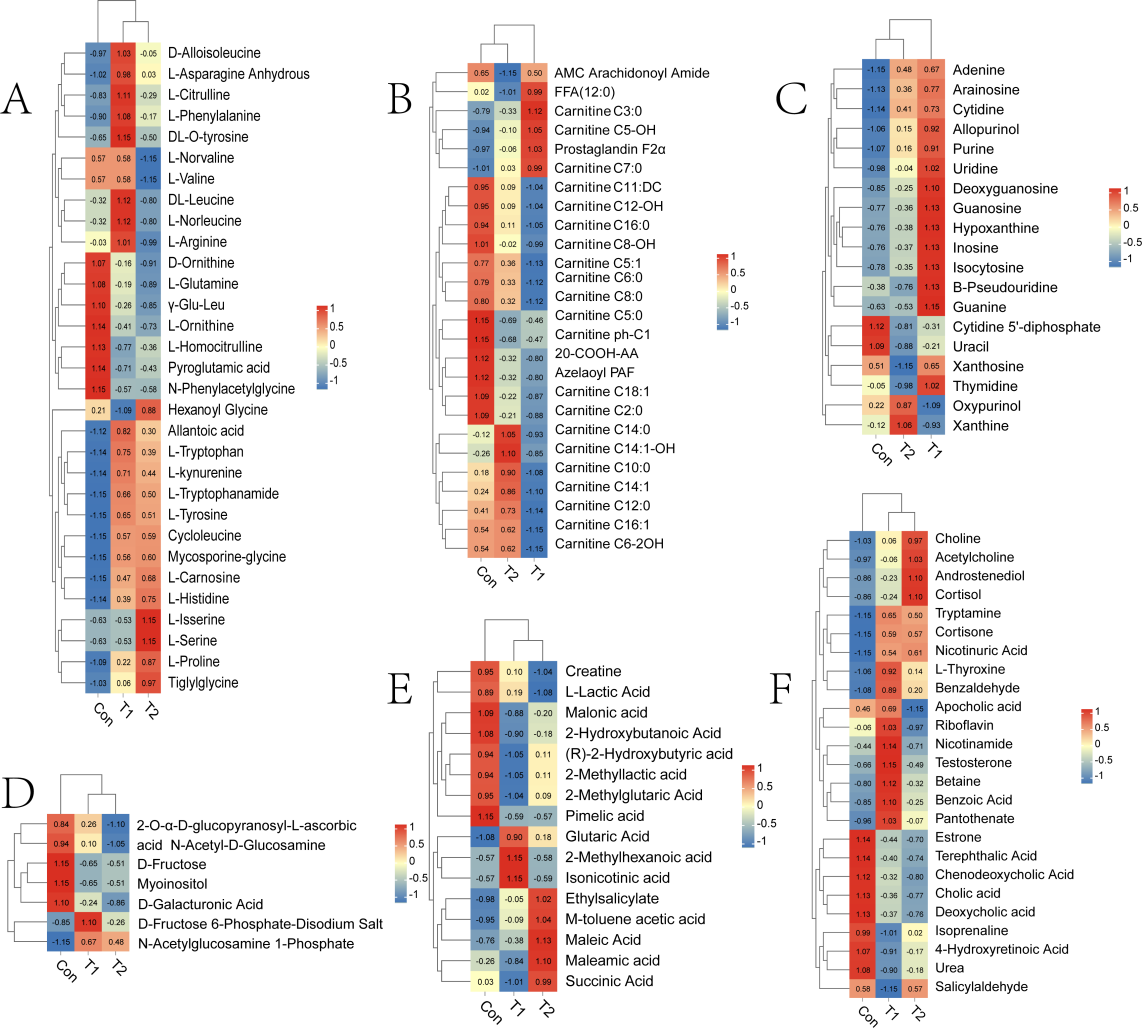
Distributions of accumulation profiles of differential metabolites detected during three experimental groups. (**A**) Amino acid and its metabolites; (**B**) FA; (**C**) nucleotide and its metabolites; (**D**) carbohydrates and its metabolites; (**E**) organic acid and its derivatives; (**F**) other metabolites.

The differential metabolites identified among the different groups primarily included amino acids and their metabolites, nucleotides and their derivatives, organic acids and their derivatives, carbohydrates and their metabolites, fatty acyls, and other compounds. To further explore the variations in these metabolites across the groups, a heat map analysis was conducted. The findings revealed a decreasing trend in carbohydrates and their metabolites, fatty acyls, bile acids, alcohols, amines, and benzene derivatives in the T1 and T2 groups compared to the Con (Fig. 8B, D, and F). Conversely, nucleotides and their derivatives, choline, tryptophan, as well as hormones and hormone-related substances exhibited an increasing trend (Fig. 8C and F). Amino acids and their derivatives, along with organic acids and their derivatives, displayed varying significance across different metabolites in the various groups due to their distinct functional roles. However, it is generally observed that the addition of herbal preparations tends to induce an upward trend (Fig. 8A and E).

**Fig 9:**
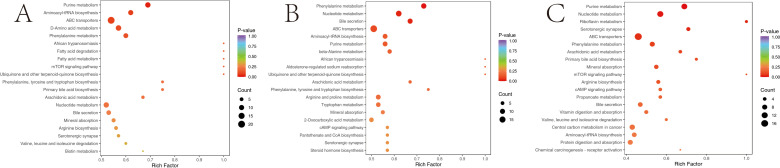
KEGG pathway enrichment analysis of serum differentially expressed metabolites. The point color represents the *P*-value, and the size of the dots represents the number of differential metabolites. (**A**) T1 vs Con; (**B**) T2 vs Con; and (**C**) T2 vs T1.

To investigate the potential metabolic pathways in sheep serum following the addition of varying proportions of herbal preparations, all differential metabolites across the different groups were aligned and scrutinized against the KEGG database to glean insights into the associated metabolic pathways. Subsequently, we conducted an analysis of the enriched pathways for the differential metabolites. The results are shown in Fig. 9. The top 5 metabolic pathways screened for differential metabolites between the T1 vs Con groups were purine metabolism, aminoacyl-tRNA biosynthesis, ABC transporters, D-amino acid metabolism, and phenylalanine metabolism (Fig. 9A). The top 5 metabolic pathways identified for the differential metabolites between the T2 vs Con groups were phenylalanine metabolism, nucleotide metabolism, bile secretion, ABC transporters, and aminoacyl-tRNA biosynthesis (Fig. 9B). The top 5 metabolic pathways screened for differential metabolites between the T2 vs T1 groups were purine metabolism, nucleotide metabolism, riboflavin metabolism, serotonergic synapse, and ABC transporters (Fig. 9C).

### Correlation analysis between rumen microorganisms and metabolome

**Fig 10:**
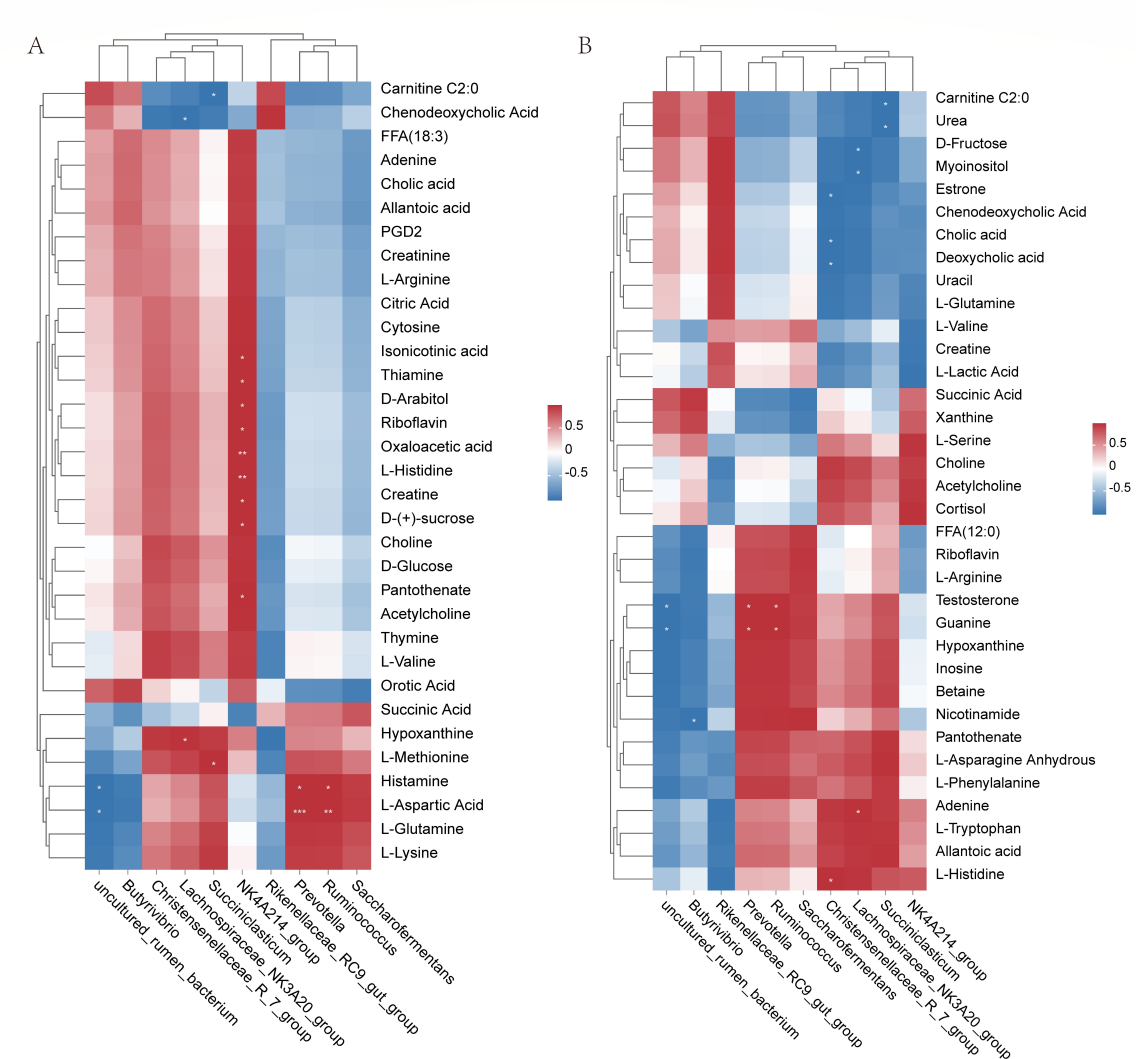
(**A**) Correlation analysis between genus-level microorganisms and rumen metabolite concentrations after addition of herbal preparations. (**B**) Correlation analysis between genus-level microorganisms and rumen metabolite concentrations after addition of herbal preparations. Each row of the graph represents a species, each column represents a metabolite, and each grid represents the Pearson correlation coefficient between an ingredient and a metabolite. Red represents positive correlation and blue represents negative correlation.

Pearson correlation analysis of rumen contents with differential metabolites and the top 10 rumen microorganisms at genus level revealed that *NK4A214_group* was positively correlated with most rumen metabolites. *uncultured_rumen_bacterium* was significantly negatively correlated with rumen metabolites histamine and L-arginine, but *Prevotella* and *Ruminococcus* were significantly positively correlated with both metabolites. *Lachnospiraceae_NK3A20_group* was found to be significantly negatively correlated with chenodeoxycholic acid. Also, *Succiniclasticum* was found to be significantly negatively correlated with carnitine C2:0 (Fig. 10A).

Pearson correlation analysis was used to correlate serum differential metabolites with the top 10 rumen contents microorganisms (genus level). The results showed that *uncultured_rumen_bacterium* was significantly negatively correlated with serum metabolites testosterone and guanine, but *Prevotella* and *Ruminococcus* were significantly positively correlated with both metabolites. *Christensenellaceae_R_7_group* was found to be significantly negatively correlated with cholic acid and deoxycholic acid. *Lachnospiraceae_NK3A20_group* was significantly negatively correlated with D-fructose and myoinositol, but it is positively correlated with adenine. *Succiniclasticum* was significantly negatively correlated with urea and carnitine C2:0 (Fig. 10B).

## DISCUSSION

In this experiment, the average daily weight gain and average daily feed intake after the addition of herbal preparations were significantly higher than Con, and the feed conversion ratio also showed a significant decrease compared to the Con (Table S3). In this study, we conducted 16S rRNA sequencing of rumen microbial populations in Hu sheep following the administration of varying dosages of herbal preparations. The relative abundance and diversity of these microorganisms serve as crucial indicators of rumen microbiota health. ACE is an index used to estimate the number of species in a community; Chao1 index is one of the measures of species richness. Shannon and Simpson indices are known to evaluate richness and evenness. Our alpha diversity analysis unveiled notable variations in rumen microbial diversity across the three experimental groups, as evidenced by differences in the Shannon index, Simpson index, Chao index, and ACE index. Specifically, the microbial diversity in the T1 and T2 groups was significantly lower than that in the Con, indicating a pronounced impact of herbal preparations on diminishing rumen microbial diversity. Moreover, the PCoA revealed further significant distinctions among the experimental groups. Previous research has consistently highlighted Bacteroidota and Firmicutes as the predominant phyla in the rumen microbiota of ruminants (24, 25). The findings of this experiment align with those of prior research. Firmicutes is mainly used to promote fiber decomposition and serves as the primary fiber-degrading bacteria in the rumen (26). On the other hand, Bacteroidetes are adept at digesting complex carbohydrates (27). Our experiment revealed a higher relative abundance of Firmicutes in the T1 and T2 groups compared to the Control group following the administration of herbal preparations, indicating a potential positive impact of these preparations on enhancing the survival and colonization of Firmicutes. At the genus level, *Prevotella* emerges as the dominant genus in the rumen, holding a significant position in rumen microbiology (28, 29), and primarily contributes to the degradation of proteins, peptides, and starches, with a role in fiber degradation as well (30). This study revealed an increase in the prevalence of *Prevotella* and *uncultured_rumen_bacterium* genera in the rumen following the supplementation of herbal preparations. Notably, the T1 group exhibited the highest relative abundance of *Prevotella*, suggesting that the inclusion of 0.5% herbal preparations facilitated the colonization of *Prevotella* in the rumen. Additionally, it is proposed that *Rikenellaceae_RC9_gut_group* may play a role in fermenting carbohydrates and proteins, potentially enhancing lipid metabolism (31). The relative abundance of *Rikenellaceae_RC9_gut_group* was significantly lower in the T1 and T2 groups compared to the Con after the administration of herbal preparations. These findings underscore the impact of nutrient composition changes on the relative abundance and diversity of rumen microbial communities.

Furthermore, the gene functions of microorganisms were predicted utilizing PICRUSt software, revealing notable distinctions in microbial functionality among the three experimental groups. Metabolism of other amino acids, glycan biosynthesis, and metabolic function were significantly higher in the T1; the results were similar to those of rumen metabolome, suggesting that the addition of 0.5% herbal preparation favored the synthesis and metabolism of amino acids. In the rumen microbiota KEGG pathways, the functions of drug resistance: antimicrobial and membrane transport were higher in the T1 and T2 groups than in the Con, suggesting that the herbal preparations may have enhanced the antimicrobial capacity of the animal organism.

To gain deeper insights into the impact of herbal preparations on rumen microorganisms, we performed metabolomics analysis. LC-MS metabolomics stands out as a comprehensive, efficient, and potent technique for assessing metabolic profiles and functional metabolites (32). In this study, we investigated the impact of herbal preparations on the rumen metabolomics of Hu sheep through metabolomic analysis. PCA results revealed a significant differentiation in the metabolite composition between the Con and the experimental group following the administration of varying proportions of the herbal preparation, indicating a substantial alteration in rumen metabolite composition due to the herbal preparation. Differential metabolite analysis demonstrated changes in the concentrations of rumen contents and serum metabolites across all three groups, potentially linked to shifts in rumen microbial relative abundance. Specifically, nucleotides and their metabolites, carbohydrates and their derivatives, organic acids, bile acids, heterocyclic compounds, as well as coenzymes and vitamins exhibited an upward trend in the T1 and T2 groups compared to the Con. Some studies have shown that amino acids and dipeptides may act as precursors for microbial protein synthesis, while carbohydrates and fatty acids can contribute to energy production for microbial protein synthesis (33). Hence, the findings of this study indicate that the supplementation of herbal preparations could enhance microbial protein content. Although microorganisms in the rumen are able to efficiently convert D-glucose into volatile fatty acids, clinical studies have shown that a significant amount of D-glucose is actually translocated directly from rumen contents to the bloodstream, suggesting that ruminal uptake of D-glucose is not insignificant (11).

The detrimental impacts of polyunsaturated fatty acids on rumen microbial communities, particularly cellulolytic bacteria and fungi, are well-documented (34). Consequently, the biohydrogenation of polyunsaturated fatty acids into fatty acids represents a crucial microbiological process in the rumen (35). Bile acids are associated with the absorption and metabolism of dietary lipids and interact with the rumen microbiome (36). Bile acids have demonstrated the ability to emulsify fat, aid in the breakdown of cholesterol and fat absorption, and enhance fat digestion by enlarging the fat surface area in contact with pancreatic lipase (37). Supplementation of high-fat diets with bile acids has been proven effective in preventing obesity development in mice (38). Furthermore, bile acids possess antiviral, antifungal, and antipyretic properties (39). The findings of this study revealed a significant increase in bile acid content in the rumen of the T2 group following the addition of herbal preparations. This suggests that the inclusion of 1.0% herbal preparations benefited lipid digestion in the rumen of Hu sheep while also enhancing the rumen’s antiviral and anti-inflammatory effects.

L-methionine serves as a precursor for other sulfur-containing amino acids and is considered an essential and limiting amino acid in the growth process of ruminants (40). In the rumen, L-methionine is primarily synthesized from glucose, inorganic sulfur, and homocysteine (41). Furthermore, L-methionine plays a crucial role in the synthesis of bacterial cell protein in the rumen (42). Catabolism of L-methionine by rumen microorganisms leads to the synthesis of amino acids and microbial proteins, which are the main sources of amino acids utilized by cells in ruminants (43). Prior studies have indicated that L-glutamine, converted to L-glutamic acid through rumen microbe-mediated hydrolysis, plays a crucial role in sustaining various essential functions. It influences microbial growth and efficiency, impacting nutrient metabolism, immune response, intestinal integrity, and the synthesis of biologically active compounds. Additionally, it serves as a potential inhibitor of amino acids, peptides, and analogs utilized by rumen bacteria (36, 44). Ruminal microorganisms are able to extensively degrade extracellular L-glutamine, hydrolyze it to L-glutamic acid and ammonia, and utilize both amino acids intracellularly for protein synthesis (45). In this study, the serum L-glutamine levels decreased following the incorporation of herbal supplements, suggesting that supplementing the diet with herbs may enhance the organism’s immune response and nutritional metabolism.

Nucleotides are raw materials for the synthesis of nucleic acids and are also involved in processes such as energy metabolism and metabolic regulation. Feed proteins are primarily absorbed by the body as amino acids after being broken down by proteases in the animal digestive tract. The rumen metabolites cytosine, xanthine, and pseudouridine nucleosides are predominantly associated with purine and pyrimidine metabolism, and fluctuations in these metabolites are related to apoptosis of rumen microorganisms (46, 47). The results of this experiment indicate that the cytosine content in both rumen and serum increased after the addition of the herbal preparation, reaching significantly higher levels than the other two groups following the addition of 1.0% herbal preparation. This may suggest that the addition of the herbal preparation is beneficial for enhancing the activity of rumen microorganisms in sheep. Choline is an important nutrient necessary for the proper functioning of the liver, muscles, and brain (48, 49). It constitutes a major component of cell and organelle membranes and is pivotal in various physiological processes, including signal transduction, DNA and histone methylation, and the formation of nerve myelin (50). Choline metabolism can biologically contribute to TCA cycling and potential gluconeogenesis. For example, choline can generate glycine and serine during methyl metabolism (51). Among the differential metabolites in the rumen and serum, the level of Choline increased with the addition of herbs to the diet, indicating that the addition of herbal preparations had a beneficial effect on the growth and homeostasis of Hu sheep. Choline can be converted to trimethylamine by certain bacteria in the Firmicutes, a process that involves the expression of the choline trimethyltransferase (cutC) gene, which is part of choline metabolism and is important for maintaining rumen health (52).

Tryptophan is an essential amino acid that is involved in protein synthesis in living organisms and is primarily derived from dietary sources (53). The small intestine absorbs proteins from food, facilitating the release of tryptophan during digestion. Subsequently, tryptophan is transported into the bloodstream through the intestinal epithelium (54, 55). Tryptophan and its metabolites, including serotonin, kynurenine, tryptamine, and various indole compounds, exhibit immunological, metabolic, and neuromodulatory functions, which are crucial for the growth and overall health of both animals and humans (56). In this experiment, the levels of L-tryptophan and L-kynurenine in the serum were significantly higher than Con, and the highest in group T1 after the addition of herbal preparations, which indicates that the addition of herbal preparations is beneficial to the healthy growth of animals. Additionally, allantoic acid is the end product of purine nucleotide metabolism in sheep Serum differential metabolite metabolism in sheep exhibited significantly higher levels of allantoic acid in the T2 group compared to the other two groups after the addition of herbal preparation. The results of serum differential metabolites showed that in pyrimidine metabolism, isocytosine, cytidine, and guanosine levels were highest in the T1 group. In the purine metabolism pathway, the levels of adenine, guanine, and hypoxanthine were notably elevated in the T1 group compared to the other two groups. This observation suggests an enhancement in purine metabolism and an acceleration in overall body metabolism in the Hu sheep following the addition of 0.5% herbal preparations to their diet.

It has been found that *Prevotella* may affect amino acid metabolism in the host (7). In this experiment, *Prevotella* was found to be positively correlated with most of the amino acid metabolites in the rumen and serum metabolite correlation analyses of the top 10 microorganisms at the rumen genus level, which is consistent with the results of the present experiment. *Prevotella* was negatively correlated with histamine (57). The present results are in contrast to this and may be due to differences at different physiological sites. *Prevotella* has been shown to play an important role in preventing diarrhea (58), reducing inflammatory and allergic diseases (59), and improving animal growth performance and meat quality (60, 61). Previous studies have found that *Lachnospiraceae_ ND3007_group* was positively correlated with methionine and lysine, the two most common limiting amino acids (AAs) in ruminants (62, 63). Lysine promotes the catabolism of proteins and amino acids by rumen microorganisms (64). *Lachnospiraceae_NK3A20_group* was positively correlated with the rumen metabolite L-lysine in this study, similar to the above results.

### Conclusions

In this study, we examined the alterations in rumen microorganisms following the supplementation of herbal preparations. We explored the variations in rumen contents and serum metabolites, as well as their interrelationships, using 16S sequencing and metabolomics approaches. Our findings revealed distinct changes in the rumen microorganism profile, encompassing shifts in species composition, relative abundance levels, and metabolic activities induced by the herbal supplements. Dominant bacteria such as Firmicutes and Proteobacteria increased in relative abundance in the rumen and were more favorable for their survival and colonization in the rumen. Furthermore, an upsurge in fermentative carbohydrate and fiber-degrading bacteria was observed. Notably, the herbal preparations induced significant modifications in both rumen contents and serum metabolite profiles. After the addition of herbal preparations, the increase in metabolites such as bile acids, L-glutamine, cytidine, and choline may play a role in the body’s antiviral and anti-inflammatory responses, nutritional metabolism, immune function, and stimulation of rumen microbial activity, with the 0.5% herbal preparation showing a more significant effect. After the addition of 0.5% herbal preparation, the increase in metabolites such as L-tryptophan and ursodeoxycholic acid in the serum is more significant, which may help to accelerate the metabolism of the organism. In addition, it was found that *NK4A214_group* was positively correlated with most rumen metabolites. *Prevotella* and *Ruminococcus* were significantly positively correlated with histamine and L-arginine. The uncultured_rumen_bacterium was significantly negatively correlated with serum metabolites testosterone and guanine, but *Prevotella* and *Ruminococcus* were significantly positively correlated with both metabolites testosterone and guanine. This study deepened our new understanding of the relationship between rumen health and nutrient metabolism of the organism. It also helps us to understand the effect of feed additives on the stability of rumen microflora and the relationship between rumen and body metabolism.

## Data Availability

The original sequencing data of rumen microbiota 16 s have been deposited into the NCBI Sequence Read Archive (SRA) database with the accession number PRJNA1141758.

## SUPPLEMENTAL MATERIAL

Supplemental files msphere.00024-25-s0001.docx Supplemental tables and figures.

ASM does not own the copyrights to Supplemental Material that may be linked to, or accessed through, an article. The authors have granted ASM a non-exclusive, world-wide license to publish the Supplemental Material files. Please contact the corresponding author directly for reuse.
