# Supplementary material for: Integrated multi-omics to elucidate the interplay between rumen microorganisms and host metabolism in Hu sheep supplemented with herbal preparations
Source: mSphere. 2025 Mar 13;10(4):e00024-25. doi: 10.1128/msphere.00024-25 (PMC12039229; doi:10.1128/msphere.00024-25)
Supplement: Supplemental files — Supplemental tables and figures. [file msphere.00024-25-s0001.docx]

## Supplementary Tables

**Supplementary Table 1** Sample sequencing data processing results statistics

| Sample ID | Raw CCS | Clean CCS | Effective CCS | AvgLen(bp) | Effective(%) |
| --- | --- | --- | --- | --- | --- |
| Con1 | 14838 | 14827 | 13339 | 1456 | 89.9 |
| Con 2 | 12565 | 12554 | 11354 | 1456 | 90.36 |
| Con 3 | 12291 | 12278 | 10983 | 1455 | 89.36 |
| Con 4 | 12501 | 12491 | 11472 | 1457 | 91.77 |
| Con 5 | 12794 | 12780 | 11693 | 1456 | 91.39 |
| Con 6 | 13285 | 13279 | 12123 | 1456 | 91.25 |
| T1-1 | 12880 | 12869 | 11818 | 1457 | 91.75 |
| T1-2 | 14638 | 14629 | 13094 | 1456 | 89.45 |
| T1-3 | 12386 | 12378 | 11054 | 1457 | 89.25 |
| T1-4 | 14718 | 14712 | 13121 | 1456 | 89.15 |
| T1-5 | 13366 | 13360 | 12107 | 1456 | 90.58 |
| T1-6 | 12877 | 12865 | 11941 | 1457 | 92.73 |
| T2-1 | 12256 | 12246 | 11304 | 1459 | 92.23 |
| T2-2 | 13505 | 13501 | 12680 | 1459 | 93.89 |
| T2-3 | 12003 | 11993 | 11328 | 1459 | 94.38 |
| T2-4 | 12516 | 12506 | 11698 | 1459 | 93.46 |
| T2-5 | 13362 | 13357 | 12822 | 1459 | 95.96 |
| T2-6 | 13159 | 13153 | 12159 | 1458 | 92.4 |

Sample ID is the name of the sample; Raw-CCS is the number of CCS identified for the sample; Clean CCS is the number of sequences identified and removed from the primers; Effective-CCS is the number of sequences filtered for length and used for subsequent analyses after removal of chimeras; AvgLen (bp) is the average sequence length of the sample; Effective (%) is the percentage of Effective-CCS to Raw-CCS. Effective-CCS as a percentage of Raw-CCS.

**Supplementary Table 2** Statistical table of species of each grade of samples

| Sample | Kindom | Phylum | Class | Order | Family | Genus | Species |
| --- | --- | --- | --- | --- | --- | --- | --- |
| Con1 | 2 | 18 | 28 | 50 | 85 | 159 | 239 |
| Con 2 | 1 | 18 | 27 | 50 | 88 | 158 | 230 |
| Con 3 | 1 | 16 | 24 | 45 | 81 | 153 | 233 |
| Con 4 | 1 | 16 | 25 | 48 | 85 | 161 | 229 |
| Con 5 | 2 | 16 | 25 | 47 | 86 | 161 | 232 |
| Con 6 | 2 | 17 | 24 | 48 | 78 | 149 | 225 |
| T1-1 | 2 | 18 | 26 | 48 | 84 | 181 | 252 |
| T1-2 | 2 | 18 | 27 | 51 | 91 | 186 | 262 |
| T1-3 | 1 | 16 | 24 | 46 | 82 | 170 | 233 |
| T1-4 | 2 | 17 | 26 | 49 | 89 | 178 | 243 |
| T1-5 | 2 | 16 | 24 | 47 | 86 | 174 | 238 |
| T1-6 | 2 | 15 | 24 | 46 | 89 | 178 | 244 |
| T2-1 | 2 | 17 | 23 | 41 | 80 | 153 | 230 |
| T2-2 | 1 | 14 | 20 | 39 | 76 | 142 | 220 |
| T2-3 | 2 | 18 | 25 | 45 | 82 | 151 | 235 |
| T2-4 | 2 | 17 | 24 | 45 | 79 | 160 | 242 |
| T2-5 | 2 | 16 | 23 | 43 | 81 | 151 | 240 |
| T2-6 | 2 | 15 | 21 | 39 | 74 | 147 | 227 |
| Total | 2 | 22 | 33 | 64 | 125 | 248 | 389 |

**Supplementary Table 3** Effects of Chinese herbal preparations on growth performance of Hu sheep

| Items | Con | T1 | T2 | *P*-value |
| --- | --- | --- | --- | --- |
| IBW/KG | 19.60±1.63 | 19.65±1.70 | 19.45±1.65 | 0.977 |
| FBW/KG | 35.73±4.08 | 38.70±2.35 | 39.69±3.12 | 0.125 |
| ADG(kg/d) |  |  |  |  |
| Days 1 to 20 | 0.17±0.05 | 0.22±0.04 | 0.22±0.04 | 0.131 |
| Days 20 to 40 | 0.21±0.04 | 0.24±0.05 | 0.20±0.06 | 0.339 |
| Days 40 to 60 | 0.24±0.07 | 0.21±0.05 | 0.25±0.08 | 0.703 |
| Days 60 to 80 | 0.23±0.06 | 0.29±0.05 | 0.26±0.06 | 0.197 |
| Days 1 to 90 | 0.18±0.03^b^ | 0.21±0.02^a^ | 0.23±0.02^a^ | 0.006 |
| DMI(kg/d) | 1.28±0.00^c^ | 1.30±0.00^b^ | 1.33±0.00^a^ | <0.001 |
| F/G | 7.32±1.35^a^ | 6.17±0.47^b^ | 5.94±0.45^b^ | 0.031 |

In the same row, values with no letter or the same letter superscripts mean no significant difference (*P*>0.05), while with different small letter superscripts mean significant difference (*P*<0.05)

**Supplementary Figure**

**
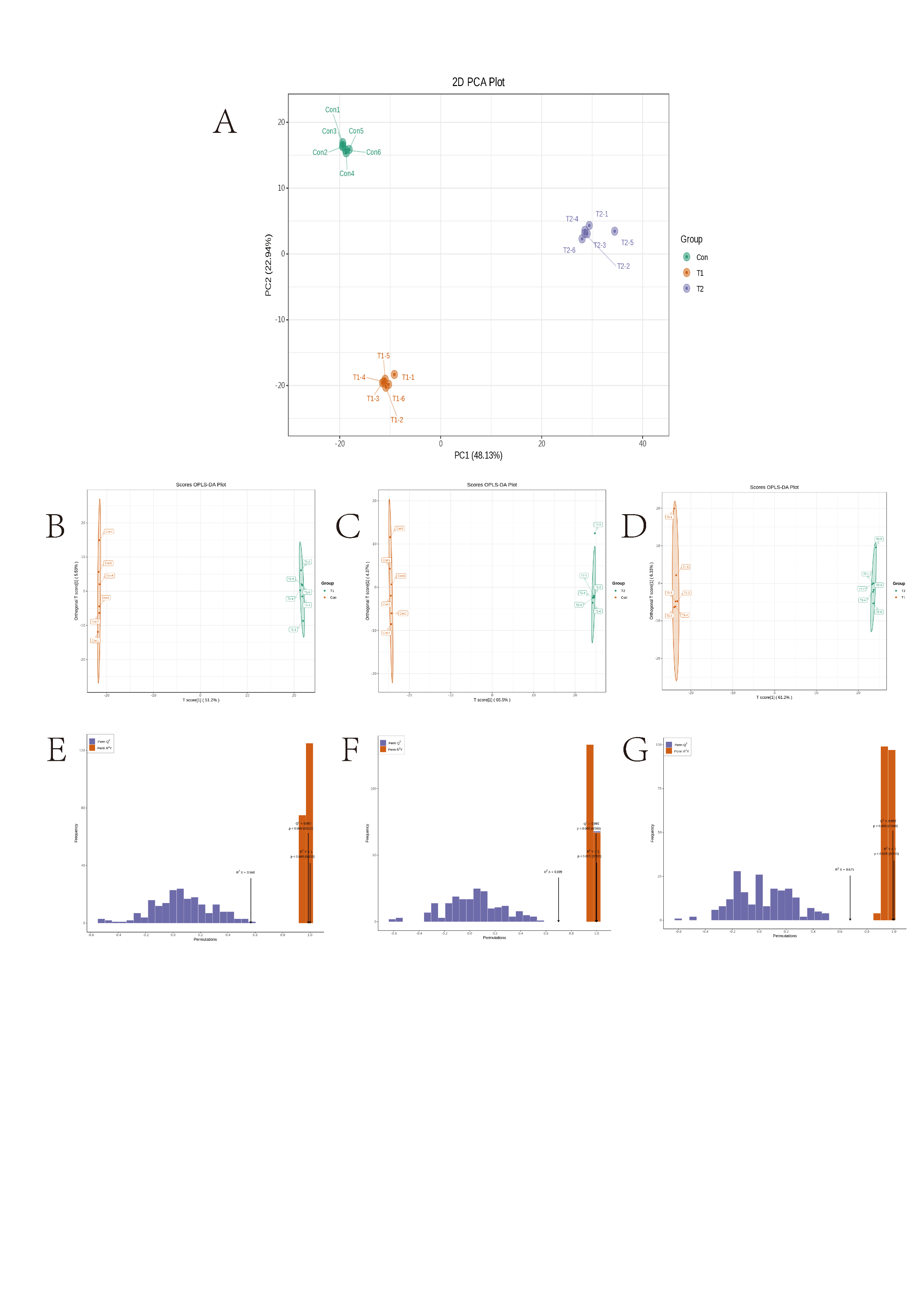
**

**Supplementary Figure 1:** Total sample of rumen PCA analysis and OPLS-DA scores plots and model validation diagrams of metabolomics data.(A) Principal component analysis of metabolomics data from three test groups of rumen contents.(B and E) OPLS-DA scores plots and model verification graph of T1 vs Con, respectively.(C and F) OPLS-DA scores plots and model verification graph of T2 vs Con, respectively. (D and G) OPLS-DA scores plots and model verification graph of T2 vs T1, respectively.

**
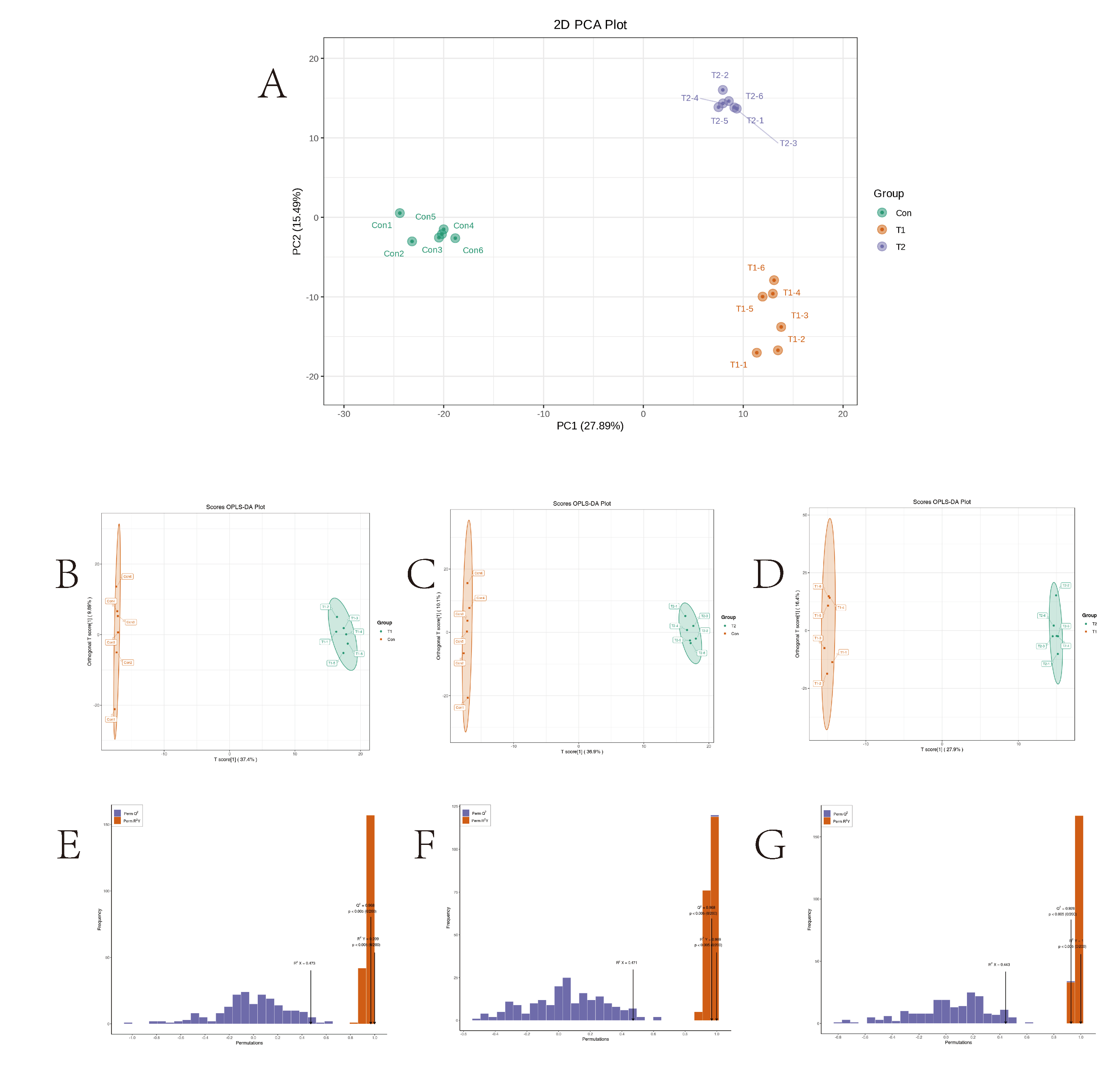
**

**Supplementary Figure 2:** Total sample of serum PCA analysis and OPLS-DA scores plots and model validation diagrams of metabolomics data.(A) Principal component analysis of metabolomics data from three test groups of rumen contents.(B and E) OPLS-DA scores plots and model verification graph of T1 vs Con, respectively.(C and F) OPLS-DA scores plots and model verification graph of T2 vs Con, respectively. (D and G) OPLS-DA scores plots and model verification graph of T2 vs T1, respectively.
